# The Exploration for an Appropriate Vacuum Level for Performance Enhancement of a Comb-Drive Microscanner

**DOI:** 10.3390/mi8040126

**Published:** 2017-04-16

**Authors:** Rong Zhao, Dayong Qiao, Xiumin Song, Qiaoming You

**Affiliations:** 1 Key Laboratory of Micro/Nano Systems for Aerospace, Ministry of Education, Northwestern Polytechnical University, Xi’an 710072, China; zhaorong@mail.nwpu.edu.cn; 2 Shaanxi Province Key Laboratory of Micro and Nano Electro-Mechanical Systems, Northwestern Polytechnical University, Xi’an 710072, China; 3 Xi’an Zhisensor Technologies Co. Ltd., Xi’an 710077, China; xiumin.song@zhisensor.com; 4 LeadMEMS Science and Technology Ltd., Xi’an 710075, China; qmyou@leadmems.com

**Keywords:** microscanner, optical scanning angle, vacuum operation, optimum pressure

## Abstract

In order to identify the influence of the vacuum environment on the performance of a comb-drive microscanner, and indicate the optimum pressure for enhancing its performance, a comb-drive microscanner fabricated on silicon-on-insulator (SOI) substrate was prepared and tested at different pressures, and the characteristics in vacuum were obtained. The test results revealed that the vacuum environment enhanced the performance in the optical scanning angle, and decreased the actuation voltage. With a 30 V driving voltage applied, the microscanner can reach an optical scanning angle of 44.3° at a pressure of 500 Pa. To obtain an enhancement in its properties, only a vacuum range from 100 to 1000 Pa is needed, which can be very readily and economically realized and maintained in a vacuum package.

## 1. Introduction

With the development of micro-electro-mechanical system (MEMS) technology, MEMS devices have been used in many fields, such as RF-MEMS, optical-MEMS, sensors, energy harvesters, and bio-MEMS. Among them, the microscanner, a promising optical-MEMS device, is widely used in LiDAR [1,2], pico-projectors [3], barcode readers [4], VR (Virtual Reality)/AR (Augmented Reality) applications [5], and so on. Mainstream actuation techniques used in microscanners are electrostatic [6], electromagnetic (EM) [7], piezoelectric [8], and electrothermal [9] actuations, and the drive forces are electrostatic forces, Lorentz or magneto-static forces, piezoelectric effects, and metal thermal effects, respectively. In the case of thermal actuation, two or more materials with different thermal expansion are used to achieve mechanical actuation. Although the thermal bimorph actuator provides a large static mechanical force at a relatively low driving voltage, the long thermal response time and non-resonant mode limit its application. Piezoelectric actuators can respond rapidly to driving signals, but the complicated fabrication of piezoelectric materials increases the difficulty in the development of these microscanners. Compared with thermal actuation and piezoelectric actuators, electrostatic and electromagnetic actuators are considered to be more suitable for microscanners because of the rapid response to the driving signal and their relatively high resonant frequency. Generally, electromagnetic actuators can offer a large driving force, but the deposited coils and permanent magnets they have result in a bulky packaging. In contrast, even though electrostatic actuators need a relatively high driving voltage, the simple and compact structure, the moderate scanning angle, and the comparatively simple fabrication process make them attract more interest in driving microscanners.

The application to display devices requires high-performance scanners, which should have high frequencies and large scanning angles to achieve good display quality. For all resonant microscanners based on different actuation mechanisms, the oscillation amplitude is determined by the input energy and loss. Air damping generates a large loss in all microscanners [10]. Especially for electrostatic microscanners, which have a structure of comb fingers and a mirror plate, they suffer from slide-film damping and squeeze-film damping. To achieve a large scanning angle, enhancing the input energy or reducing the loss energy (mainly caused by air damping) is expected. Therefore, two methods have been used to meet the requirement of a low driving voltage and a large scanning angle in its application: adopting hybrid actuation mechanisms to drive the microscanner [11] (enhancing input) and vacuum packaging [3,12] (reducing loss). However, the hybrid actuation combined electrothermal actuators and electromagnetic actuators, which is complex in fabrication and control. As for vacuum packaging, it would not change the device’s structure. Additionally, it is effective for decreasing the driving voltage [13] and promoting the scanning angle and quality factor [14,15]. Therefore, vacuum packaging seems to be an ideal way to enhance the performance of the microscanner. Compared to other types of actuators, the amplitude of the electrostatic actuator is more obviously affected by vacuum packaging.

As stated by the description before, a vacuum-packaged electrostatic microscanner is appropriate for display devices. However, the earlier results showed that the oscillation frequency range has been decreased at high vacuums levels [13,14,15], which leads to an instability of the oscillation frequency, especially when temperature varies [16]. Although many reports agree that a high-level vacuum package will enhance the scanning angle of the microscanner, the narrow frequency range, the difficult sealing technology, and the high cost cannot be ignored. Furthermore, thermal management is a critical issue in high-vacuum packaging. Without a convection medium, such as air, to assist in heat dissipation, the thermal energy induced in the mechanical movement will accumulate on the device and affect its mechanical properties. Furthermore, the dynamic response and reliability could worsen, even though the vibration angle might increase. In order to obtain a relatively good performance, considering the leverage on stability, as well as on cost, in this paper we investigated the detailed characteristics of the microscanner in a vacuum to explore the appropriate vacuum level as used for packaging. The properties of the frequency response, excitation voltage, and the change of the stable and unstable regions are studied in atmospheric air and vacuum.

## 2. Materials and Methods 

### 2.1. Device Description

**Figure 1:**
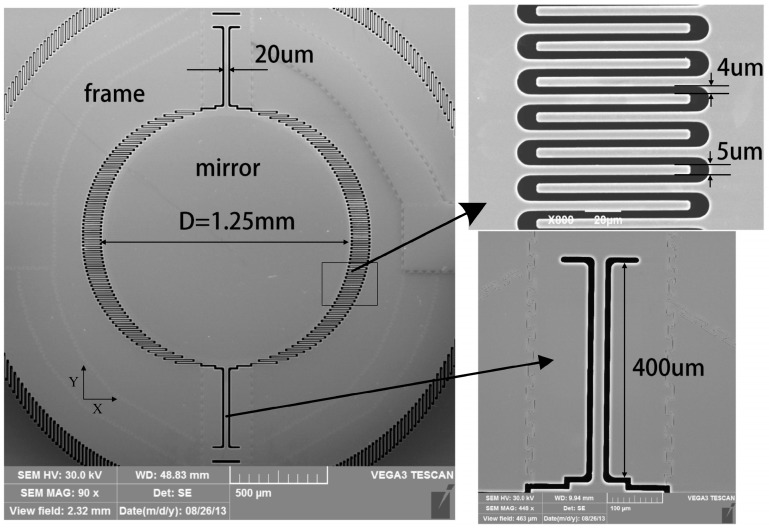
The scanning electron micrograph of the micro mirror structure. (**Top right**) The structure of comb finger; and (**bottom right**) the larger version of the torsion beam.

**Figure 2:**
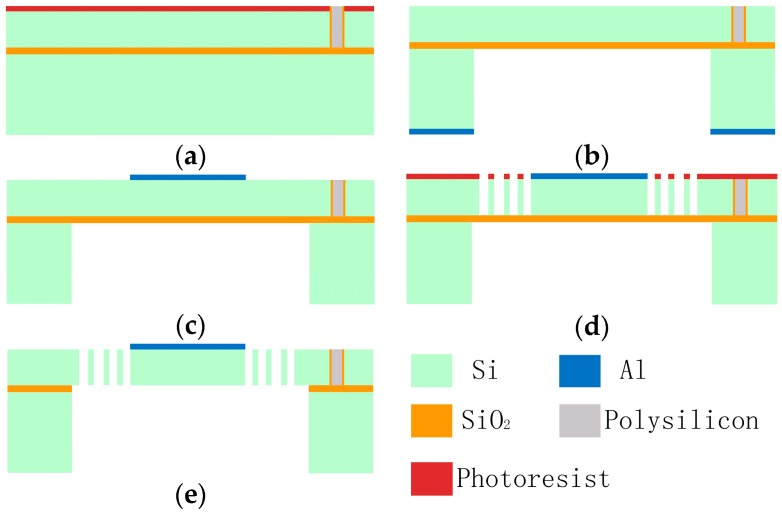
Fabrication process of the microscanner. (**a**) Isolation trench fabrication; (**b**) the back is etched to form the back cavity; (**c**) sputtering of the reflective film; (**d**) the front is etched to form the comb and structure; and (**e**) removal of the buried oxide to release the movable structures.

The microscanner used in the vacuum test consists of a reflection mirror, a movable frame, and an electrostatic comb-drive actuator. The mirror and frame are connected with torsion beams. The scanning electron micrograph of the microscanner is shown in Figure 1. It is fabricated on SOI wafers with the process flow illustrated in Figure 2.

The fabrication process begins with an SOI wafer. A 454 μm-thick SOI wafer, which has a 4-μm-thick buried oxide layer and a 50-μm-thick device layer, is used. The first step of the process is making an isolation trench by photolithography and inductively-coupled plasma (ICP) etching with a polymer photoresist mask, and then the trench is filled with polysilicon by low pressure chemical vapor deposition (Figure 2a). Secondly, the photoresist polymer film is removed by O_2_ plasma and the polysilicon over the wafer was then removed by chemical-mechanical polishing before depositing an aluminum film over the handle layer. After that, aluminum is deposited on the back of the substrate and patterned by wet etching. Then, ICP etching is used to form the back cavity using the aluminum mask (Figure 2b). Thirdly, the residual aluminum mask film on the substrate is removed and, to obtain the reflective film, aluminum is sputtered onto the device surface (Figure 2c). Fourthly, the photolithography proceeds and the device layer is etched to form the device structures, including the inner mirror, comb fingers, and outer frame (Figure 2d). Finally, the remaining photoresist polymer is cleaned, and the buried oxide underneath the moving parts is removed in a HF solution to form the movable mirror structure (Figure 2e).

The torsion equation of motion of the microscanner is given by:

(1) $$T_{e}=I\ddot{\theta }+b\dot{\theta }+K_{\theta }\theta$$

where *θ* is the torsion angle of the microscanner, *b* is the damping coefficient, *T* is the torque applied, *K_θ_* is the elastic coefficient, and *I* is the moment of inertia, respectively. 

**Figure 3:**
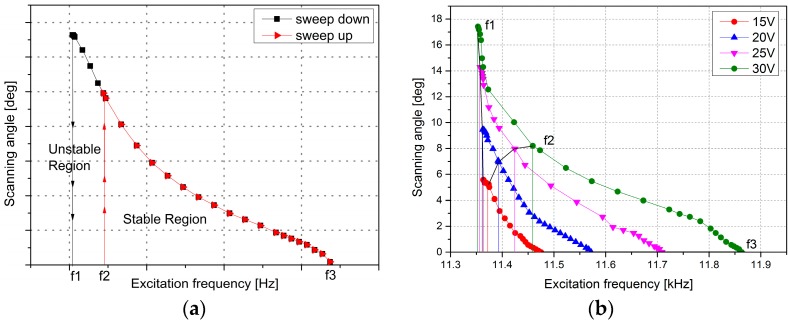
(**a**) Microscanner frequency response curves. (**b**) The frequency response curves with different driving voltages at atmospheric pressure of the microscanner used in this work.

The equation is a typical parametrically-excited system; thus, the comb-drive microscanner is a typical nonlinear parametric system [17]. The performance characteristics of the microscanner sweeping from different directions of frequency will demonstrate a hysteresis effect, and there will be two hopping frequencies, *f*_1_ and *f*_2_ (*f*_2_ > *f*_1_), in the frequency response curve, as shown in Figure 3a. The interval between the two jump frequencies *f*_1_ and *f*_2_ is the unstable region [18]. In the unstable region, oscillations can only be observed if the external frequency is swept down to this region from *f*_2_, but when the frequency is swept up from *f*_1_, no oscillation occurs. In contrast, in the stable region, the oscillation happens irrespective of the sweep direction.

**Figure 4:**
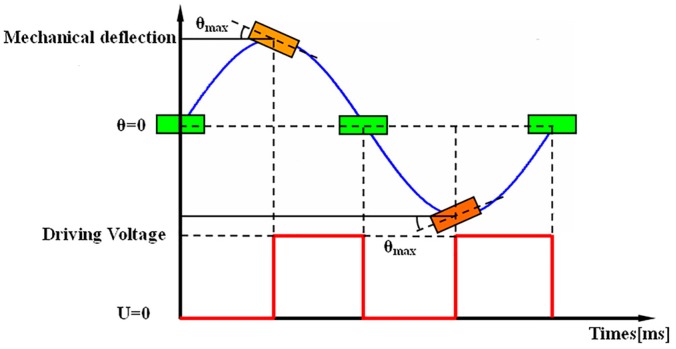
Driving principal of the microscanner. The frequency of the driving signal is double that of the microscanner.

The driving principle of the electrostatic microscanner is shown in Figure 4. A square wave is commonly used as the excitation signal, and the switch-off time of the driving signal coincides with the moment the mirror plates pass the resting position. When the voltage pulse ends, the plate swings back by inertia. The movement is then only guided by mechanical properties (spring stiffness and the moment of inertia of the plate). The next pulse starts at maximum deflection and ends again at the rest position. The microscanner can only work in resonance, and to achieve the largest scanning angle at a fixed driving voltage, it needs to be excited by a signal with a frequency near twice its natural frequency of the torsion mode [4]. Other signals, like triangular waves, sawtooth waves, or sine waves, can also be employed as the excitation signal for the microscanner, and the oscillation amplitudes of the microscanner excited by them are slightly smaller than the amplitude excited with a square wave at the same applied voltage [6]. In this work, the microscanner was excited by a square wave at 30 V.

The microscanner will reach the maximum scanning angle *θ*_0*r*_ when the resonance frequency is its natural frequency *ω_r_*, with the following equation [18]:

(2) $$\theta_{0r}=T_{0}/\omega_{r}b$$

where *T*_0_ is the electrostatic torque generated by the comb fingers, and *b* is the damping coefficient. The optical scanning angle is a very important parameter to the microscanner, which directly determines the size of the image and the required driving voltage range. One of the properties to indicate superior performance of the device is that the device can obtain a larger scanning angle with a smaller actuation voltage. From Equation (2), it is showed that one way to enhance the scanning performance is to lower the damping coefficient.

Air damping is a significant factor influencing the performance of the microscanner [10]. Thus, theoretically, in a vacuum environment the device can reach a larger scanning scale than that at atmospheric pressure with the same actuation conditions.

### 2.2. Principles

For the resonant electrostatic microscanners driven by a square wave, the input energy comes from the driving voltage. At the rest position, the total energy provided by square wave is expressed as:

(3) $$E=C_{0}V^{2}$$

where *C*_0_ is the capacitance at the rest position (the maximum capacitance) and *V* is the voltage. In an oscillation cycle, the loss energy of the electric field is calculated by the following equation:

(4) $$\Delta E^{R}=\left\{ \begin{array}{l} C_{0}V^{2}\frac{\theta_{0}}{\theta_{c}},\theta_{0}\leq \theta_{c}\\ C_{0}V^{2},\theta_{0}>\theta_{c} \end{array}\right.$$

where *θ*_0_ is the torsion angle at voltage *V*, and *θ_c_* is the angle where the fixed and movable comb fingers have no overlap. Since the quality factor *Q* is defined as the ratio between the kinetic energy of the mirror and the loss energy, *Q* is obtained by the equation:

(5) $$Q=\frac{\pi I\omega^{2}{\theta_{0}}^{2}}{\Delta E^{R}}$$

where *ω* is the oscillation frequency. In a low-damping system, the quality factor is the amplitude response at the resonant frequency, which can be estimated as:

(6) $$Q=\frac{I\omega }{b}$$

From the above equations, the relationship between the voltage and the torsion angle can be roughly calculated as:

(7) $$\theta_{0}=\left\{ \begin{array}{l} \frac{C_{0}V^{2}}{b\pi \omega \theta_{c}},\theta_{0}\leq \theta_{c}\\ V\sqrt{\frac{C_{0}}{b\pi \omega }},\theta_{0}>\theta_{c} \end{array}\right.$$

A microscanner with large scanning angle and a low driving voltage is expected, which means a large ratio of *θ*_0_/*V* is needed. Generally, the situation of *θ*_0_ > *θ_c_* is considered; thus, the relationship between *θ*_0_/*V* and *b* is expressed below: because the damping coefficient *b* is greater than zero, *θ*_0_/*V* is monotonically decreased with *b*.

(8) $$\frac{\theta_{0}}{V}=\sqrt{\frac{C_{0}}{b\pi \omega }},\theta_{0}>\theta_{c}$$

Equation (8) shows a nonlinear relationship between *θ*_0_/*V* and *b*; as *b* is decreased very low, the increase of *θ*_0_/*V* becomes imperceptible. Thus, an extreme vacuum degree may be unnecessary because the vibration angle would approach a constant value behind some certain vacuum degrees. For this reason, a balance between the vacuum level and the packaging cost needs to be explored.

**Figure 5:**
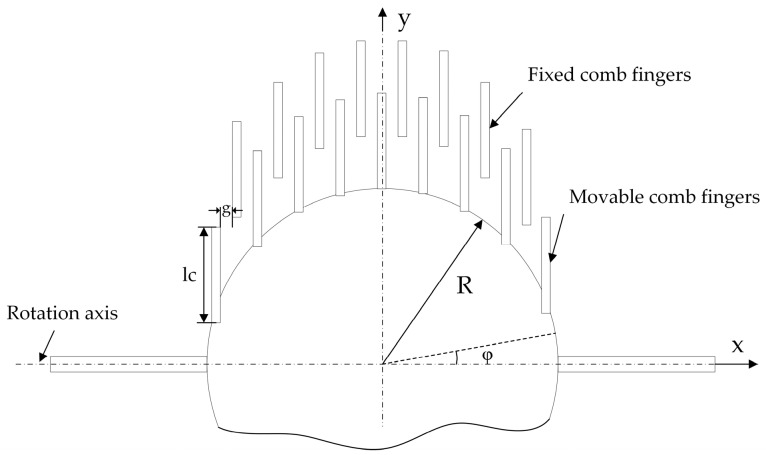
The geometry of the microscanner for calculations.

In the case of a high vacuum (less than 1000 Pa), collisions with gas particles are the dominant damping mode, and the interaction between gas molecules is neglected [19]. The damping force is generated by the interaction between gas molecules and moving structures, and this includes the torque from the mirror plate and comb fingers. Figure 5 shows the schematic diagram of the microscanner used in this work. The torque generated by the pressure difference between the front and back surfaces of the mirror plate is estimated as [14]:

(9) $$T_{plate}=\frac{\pi hR^{3}P_{i}\dot{\theta }}{c}\left\{\frac{R}{4h}\left[(2-\sigma_{n})\cdot \left(\frac{2}{\sqrt{\pi }}+1\right)+\sigma_{n}\sqrt{\frac{\pi T_{w}}{T_{i}}}\right]+\frac{1}{\sqrt{\pi }}\sigma_{t}\right\}$$

where *h* is the thickness of the mirror plate, *R* is the radius of the circular mirror plate, *P_i_* is the environment pressure, $\dot{\theta }$ is the angular velocity (*dθ*/*dt*), *c* is the thermal velocity of the gas molecules, *σ_n_* and *σ_t_* are the normal and tangential accommodation coefficients, respectively, *T_w_* is the wall temperature, and *T_i_* is the ambient temperature. The value of *c* is calculated from the gas molecules *m*, the ambient temperature *T_i_*, and the Boltzmann constant *k_B_* as:

(10) $$c=\sqrt{2k_{B}T_{i}/m}$$

In addition, the torque generated by the molecular collisions on the comb fingers is calculated by the shear stress along the outside edges *τ_sidewall_* and the front and rear edges *τ_edge_* [19]. The shear stresses of the comb fingers are obtained by:

(11) $$\tau_{sidewall}=N\frac{l_{c}\sigma_{t}P_{i}\dot{\theta }}{2c\sqrt{\pi }},\tau_{edge}=N\frac{(l_{c}+R\sin\varphi )\sigma_{t}P_{i}\dot{\theta }}{c\sqrt{\pi }}$$

Then the torque *T_comb_* is given by:

(12) $$T_{comb}=\frac{N\cdot 4h}{2c\sqrt{\pi }}\int_{0}^{\pi /2}(3l_{c}\sigma_{t}P_{i}\dot{\theta }+2R\sin\varphi \sigma_{t}P_{i}\dot{\theta })R\sin\varphi d\varphi =\frac{2Nh\sigma_{t}P_{i}\dot{\theta }}{c\sqrt{\pi }}(3l_{c}R+\frac{\pi }{2}R^{2})$$

In the above equations, the parameters not mentioned are denoted in Figure 5. Thus, the total damping torque *T* can be obtained by:

(13) $$\begin{array}{l} T=T_{plate}+T_{comb}=\frac{\pi hR^{3}P_{i}\dot{\theta }}{c}\left\{\frac{R}{4h}\left[(2-\sigma_{n})\cdot \left(\frac{2}{\sqrt{\pi }}+1\right)+\sigma_{n}\sqrt{\frac{\pi T_{w}}{T_{i}}}\right]+\frac{1}{\sqrt{\pi }}\sigma_{t}\right\}\\ +\frac{2Nh\sigma_{t}P_{i}\dot{\theta }}{c\sqrt{\pi }}(3l_{c}R+\frac{\pi }{2}R^{2}) \end{array}$$

Concerning an isothermal system (*T_w_* = *T_i_*), full momentum accommodation (*σ_n_* = 1, *σ_t_* = 1), the total torque *T* generated by the gas collisions is simplified to:

(14) $$T=P_{i}\dot{\theta }\left[\frac{\sqrt{\pi }R^{4}}{4c}\left(2+\pi +\sqrt{\pi }+4h\right)+\frac{2Nh}{c\sqrt{\pi }}(3l_{c}R+\frac{\pi }{2}R^{2})\right]$$

Since *T* = *b* × *dθ*/*dt*, and the quality factor *Q* at high vacuum is *Q* = *Iω*/*b*, the quality factor in a high vacuum *Q_v_* is expressed as:

(15) $$Q_{v}=I\omega /P_{i}\left[\frac{\sqrt{\pi }R^{4}}{4c}\left(2+\pi +\sqrt{\pi }+4h\right)+\frac{2Nh}{c\sqrt{\pi }}(3l_{c}R+\frac{\pi }{2}R^{2})\right]$$

In the case of a low vacuum (from 1000 Pa to 10^5^ Pa), the friction damping generated by the viscous flow of ambient air becomes important. The quality factor in ambient air *Q_a_* is calculated by Equation (5); thus, the loss energy is acquired.

The loss energy contains the loss of the mirror plate and comb fingers, which is estimated by [14,20]:

(16) $$L_{plate}=\pi^{2}\omega hR^{3}{\theta_{0}}^{2}{\left(\frac{\omega \rho \eta }{2}\right)}^{1/2}\left(1+\frac{R}{2h}\right)$$

(17) $$L_{comb}=\frac{2N\pi h{\theta_{0}}^{2}\eta_{eff}\omega }{3g}\left(3l_{c}R+\frac{\pi }{2}R^{2}\right)$$

where *ρ* is the air density and *η* is the dynamic viscosity of air in Nsm^−2^. At room temperature (300 K), the dynamic viscosity of air is 18.714 × 10^−6^ Nsm^−2^. The *η_eff_* is the effective dynamic viscosity of air, valued as *η*/(1 + 9.658 *K_n_*^1.159^), where *K_n_* is the Knudsen number [15].

Now, the quality factor in low vacuum is expressed as:

(18) $$Q_{a}=\frac{I\omega }{\pi^{2}hR^{3}{\left(\frac{\omega \rho \eta }{2}\right)}^{1/2}\left(1+\frac{R}{2h}\right)+\frac{2N\pi h\eta_{eff}}{3g}\left(3l_{c}R+\frac{\pi }{2}R^{2}\right)}$$

The equations above roughly analyzed the influence of air damping. Reviewing the analysis process, the impact of the vacuum is discussed in high vacuum (less than 1000 Pa) and in low vacuum (from 1000 Pa to 10^5^ Pa) separately, owing to the different damping effect in each region. Therefore, the obtained equations can be useful for the rough explanation of the experimental results.

### 2.3. Experimental Procedure

To investigate the characteristics of the fabricated microscanner in a vacuum, a laser triangulation method is used in this paper, which includes changing the voltage and the pressure, testing the scanning amplitude, and transforming that into the optical scanning angle [21].

The specific steps are as follows: Firstly, the microscanner was mounted on a support base in the vacuum chamber, and illuminated by a 532 nm green semiconductor laser, while the reflected light was received by the indicated screen. Then, the AC excitation signal was applied to the microscanner to make it deflect at a certain frequency. Since the naked eye cannot distinguish the scanning spot, the trail of the reflected laser spot will produce a scanning line, which can be measured and used to calculate the optical scanning angle using the following equation:

(19) $$\theta =\arctan\left(\frac{H+L/2}{S}\right)-\arctan\left(\frac{H-L/2}{S}\right)$$

where *θ* is the optical scanning angle of the mirror; *L* is the length of the laser scanning line after scanning; *H* is the distance between the fixed center point of the scanning line and the datum point; and *S* is the distance between the scanning mirror device and the receiving screen, respectively.

**Figure 6:**
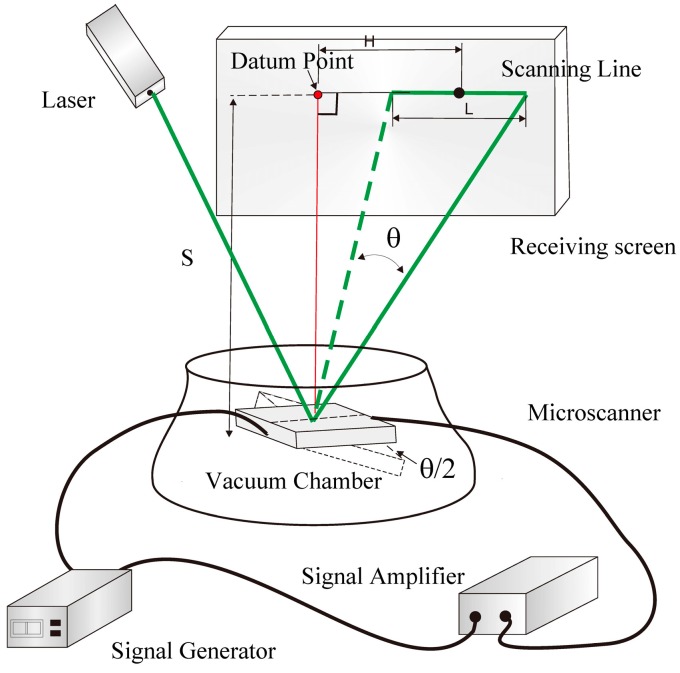
Schematic of the setup used in the vacuum test.

The measurement setup of the experiment is shown in Figure 6. When the excitation voltage and pressure are determined, the amplitude is tested through changing the frequency of the microscanner, and the corresponding amplitudes are measured and recorded. In this paper, two types of experiments were implemented. The first was carried out by sweeping the excitation frequency under given voltages and pressures to obtain the scanning angle *θ_f_*. The second was carried out by sweeping the pressure under given voltages and frequencies to obtain the maximum scanning angle *θ_p_*.

**Figure 7:**
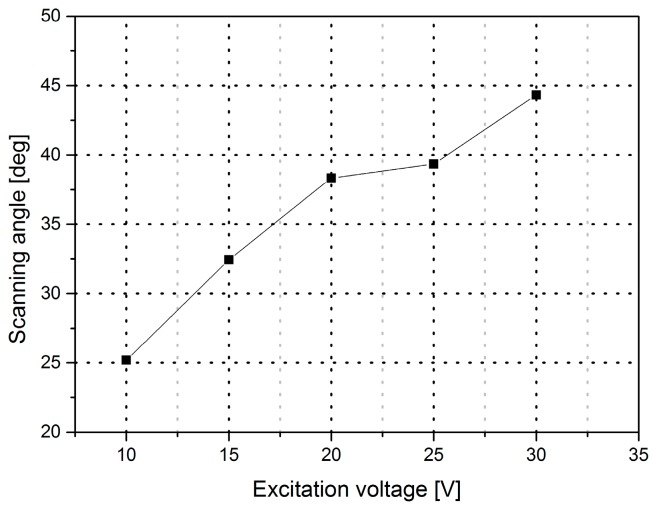
The maximum scan amplitude vs. excitation voltage.

According to the positive correlation between the excitation voltage and the optical scanning angle shown in Figure 7, this experiment was performed to test microscanners at different excitation voltages, starting at 10 V, and ending at 30 V under different pressures.

## 3. Results

### 3.1. Time Response in a Vacuum

**Figure 8:**
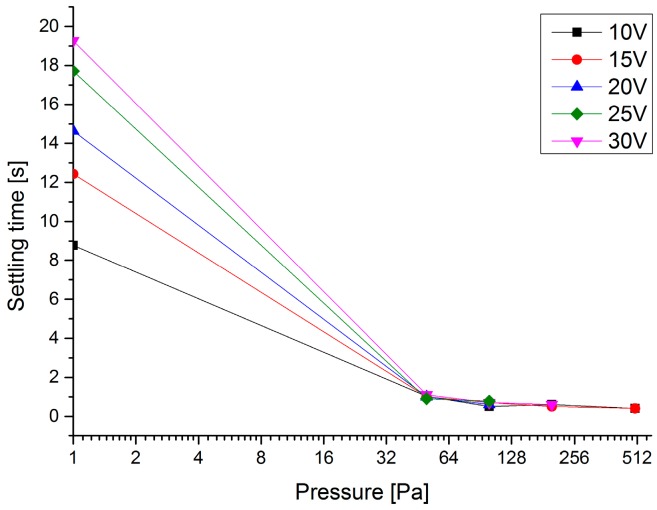
The settling time required for the microscanner to stop versus the pressure at different excitation voltages. The lower the pressure is, the longer time that is needed for the microscanner to stop.

In a vacuum, when the excitation output signal is turned off, the scanning mirror does not immediately stop because, as the pressure reduces, the air damping decreases. The settling time in mode switching was tested in the experiment. Figure 8 shows the results of the settling time versus pressure at different excitation voltages. The settling time of the microscanner from dynamic to static states at 1 Pa is more than 10 s, longer than the time at atmospheric pressure. When the pressure is higher than 100 Pa, there is hardly any settling time from oscillation to stop states, and the settling time at higher pressure is less than 0.41 s.

The settling time is high in the case of the vacuum-packaged microscanner compared to the non-vacuum-packaged microscanner. The dynamic motion of a mechanical system is affected by the sum of the structural damping and the damping of the surrounding medium in which the structure moves. Therefore, the system takes a longer time to settle in a vacuum because of the absence of air damping.

### 3.2. The Minimum Actuation Voltage in a Vacuum

**Figure 9:**
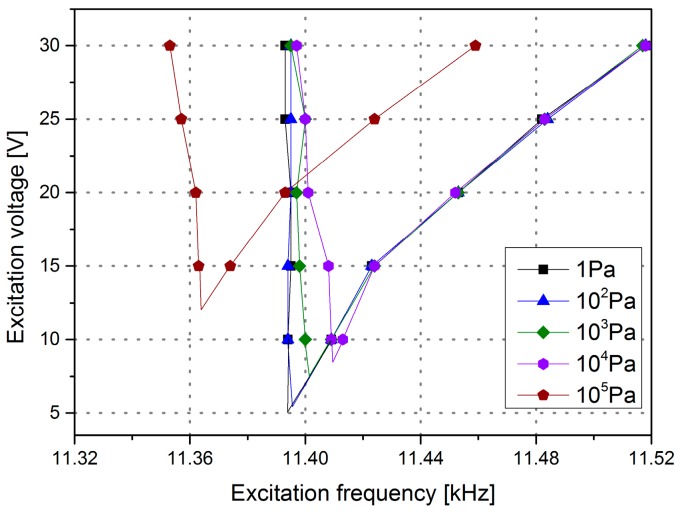
The excitation frequency versus the excitation voltage at different pressures.

For the microscanner, a low actuation voltage is expected. A small required driving voltage means a larger driving voltage range that the device can operate in and lower energy consumption. In this experiment, the frequency versus actuation voltage was tested. Figure 9 shows that the test curve is similar to the macroscopic “tongue” shape. The “tip of the tongue” part represents the minimum voltage to drive the microscanner, and the driving frequency is applied irrespective of the sweep direction. The minimum driving voltage of the mirror is lower than 8 V in a vacuum environment at a pressure ranging from 1 to 1000 Pa. In contrast, the required driving voltage at atmospheric pressure is 12.5 V.

### 3.3. The Range of Excitation Frequency in Vacuum

**Figure 10:**
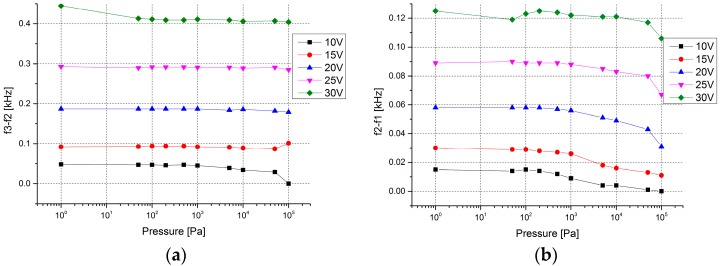
The change with pressure of the stable region and the unstable region. (**a**) The range change of the stable region. (**b**) The range change of the unstable region.

In a vacuum environment, Figure 10 shows that the range of the stable and unstable regions have been slightly influenced by the change of pressure, but it is broader than that at atmospheric pressure with the same excitation voltages applied. For the unstable region, when the pressure is higher than 1000 Pa, the range of the region becomes narrower with the increasing pressure.

### 3.4. Characteristics in the Stable Region

**Figure 11:**
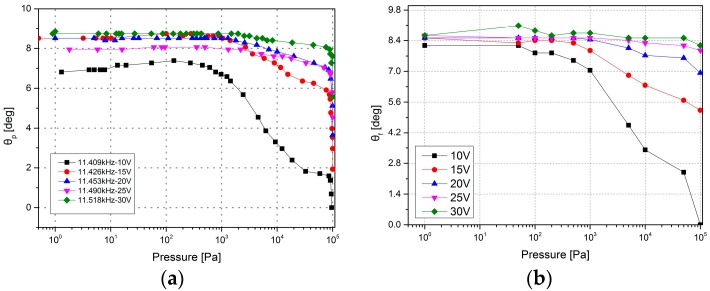
The optical scanning angle vs. pressure in the stable region. (**a**) The optical scanning angle vs. pressure with a fixed excitation frequency under different actuation voltages. (**b**) The maximum optical scanning angle vs. pressure under different actuation voltages.

In the stable region, as shown in Figure 11, the optical scanning angle has a negative correlation with pressure. Both the maximum optical scanning angle (*θ_p_*) and the scanning angle at fixed frequencies (*θ_f_*) have an improvement at lower pressure. Figure 11a shows the results of the tests under fixed frequencies: by applying the actuation voltage of 30 V, the scanning angle increased from 5.57° at atmospheric pressure to 8.74° at 500 Pa, raised by 56.96%; and with the voltage of 15 V applied, the angle increased by 352.13% from 1.93° to 8.74°. Figure 11b shows the change of the maximum optical scanning angle related to the pressure and actuation voltage. When the pressure ranges from 1 to 100 Pa, nearly all of the maximum scanning angles are above 8°, and with the decrease of the vacuum level, the influence of the actuation voltage on the scanning angle rises. The percentage of the change is inversely proportional to the actuation voltage. Here, a plateau zone has been found in Figure 11, ranging from 1 to 1000 Pa. When the vacuum level is in this zone, the microscanner will have an economical and expected scanning angle with the designed actuation voltage in the stable region.

### 3.5. Characteristics in the Unstable Region

**Figure 12:**
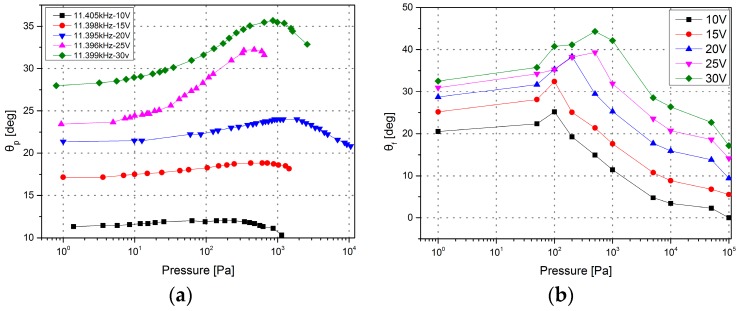
Theoretical scanning angle vs. pressure in the unstable region. (**a**) The optical scanning angle vs. pressure with a fixed excitation frequency under different actuation voltages. (**b**) The maximum optical scanning angle vs. pressure under different actuation voltages.

In the unstable region, Figure 12 shows that the optical scanning angle does not change monotonically with the pressure. When the pressure is lower than a critical value *P_c_* (*P_c_* ranges from 100 to 1000 Pa, and is dependent on the excitation voltage), the optical scanning angle increases with the increasing air pressure, but when the pressure is higher than *P_c_*, the optical scanning angle decreases with the increasing pressure. There is a peak value in the maximum optical scanning angle vs. pressure curve, and the higher the voltage is, the sharper the peak. Figure 12b represents the change of the maximum optical scanning angles in accordance with the change of pressure. At pressure *P_c_*, the maximum optical scanning angle is 44.32° when 30 V is applied, 38.34° when 20 V is applied, and 32.44° when 15 V is applied, and at atmospheric pressure, the angles are 17.16°, 9.42°, and 5.57°, respectively.

Vacuum packaging is typically reasonable to increase the scanning angle of the microscanner working in the unstable region, and only a vacuum level ranging from 100 to 1000 Pa is needed to obtain the maximum optical scanning angle. This kind of vacuum level is readily achieved in vacuum packaging and is also economical to be maintained over the lifetime of the device.

## 4. Discussion

The response time, the minimum actuation voltage, the optical scanning angle, and the range of the stable and unstable regions are indicators of the property values of the microscanner. An expected microscanner needs to have a larger projection scale, lower power consumption, and faster settling time. One method to optimize the performance and lower the power required is to reduce the air damping inside the packaged microscanner. According to the experiment results, at a pressure of 500 Pa, the maximum optical scanning angle is enhanced from 17.16° to 44.32°. From Figure 11 and Figure 12, a plateau zone ranging from 100 to 1000 Pa has been obtained. In this zone, the behaviors of the scanning angle in both stable and unstable regions have a larger promotion than at atmospheric pressure. Additionally, the high vacuum also decreases the minimum actuation voltage from 12.5 V at atmospheric pressure to below 8 V at a pressure lower than 1000 Pa. A high vacuum level has a significant influence on the settling time: greater than ten seconds is needed for the microscanner to settle down at 1 Pa. However, at a pressure higher than 100 Pa, the settling time can be neglected. 

**Figure 13:**
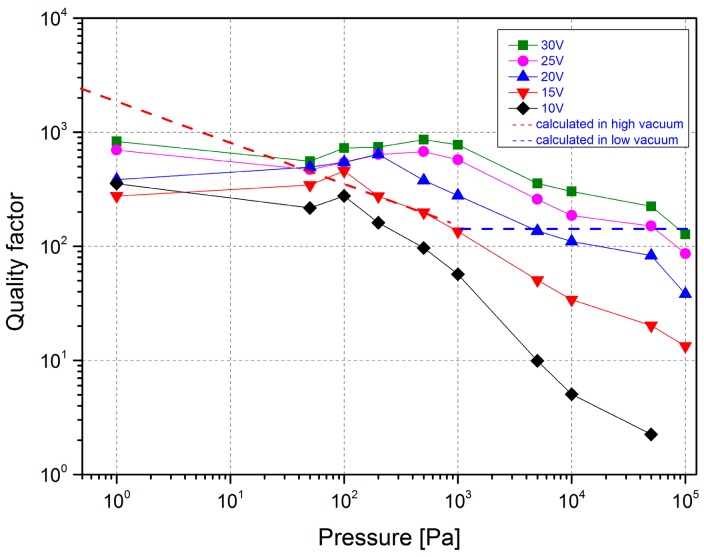
Quality factors of the microscanner measured at different driving voltages and calculated as a function of pressure.

On the other hand, the quality factor *Q* of the microscanner has been calculated from the experiment results (*θ* and *ω*) at different driving voltages, using Equations (15) and (18), shown in Figure 13. The measured values of *Q* are positively correlated with the driving voltages, but negative with the pressure. Additionally, the quality factor of the microscanner with a low driving voltage is more sensitive to pressure: the value of *Q* increases by about two orders of magnitude when compared with that in atmospheric air in the case of a 10 V driving voltage. With the pressure decreasing from 10^5^ to 1000 Pa, the values of *Q* increase. The growing trends start to stabilize from at 1000 Pa and below. In comparison with the measured values, the theoretical *Q* values are calculated by Equations (15) and (18) with an average energy input in the high and low vacuum, respectively. Although the theoretical values of *Q* obtained by the equations are underestimated, the same segmentation point of the trend (1000 Pa) is valuable. The curve slope in the range from 100 Pa to 1000 Pa roughly fits with the measured values; thus, the principal based on molecular collision can explain the damping mechanism. When the pressure is lower than 10 Pa, the measured results show independence with pressure, and the loss is mainly caused by thermal dissipation or intrinsic damping.

Above all, the pressure of maintaining optimal properties is a medium vacuum level in the experiment, ranging from 100 to 1000 Pa, and providing a medium vacuum to meet the large-sized projection is particularly easy and economical to implement in an industrial package.

## 5. Conclusions

This paper emphatically expounds the performance testing of a microscanner at different pressures to explore the optimum pressure in improving the performance of the device. The test results revealed that the vacuum package is especially reasonable to enhance the scanning angle for the microscanner working in the unstable region. The device can achieve a 44.3° optical scanning angle at 30 V, requiring almost no settling time at pressures ranging from 100 to 1000 Pa, and the performance of the microscanner under these conditions is improved. Thus, a larger projection size is achieved in a medium vacuum environment to satisfy the demands of business projection, which is very readily and economically realized and maintained in the vacuum package. The results of the microscanner experiment in vacuum provide a reference for the vacuum packaging.
